# (*E*)-2-[(4-Fluoro­phen­yl)imino­meth­yl]-5-methoxy­phenol

**DOI:** 10.1107/S1600536810000474

**Published:** 2010-01-09

**Authors:** Çiğdem Albayrak, Arzu Özek, Başak Koşar, Mustafa Odabaşoğlu, Orhan Büyükgüngör

**Affiliations:** a Faculty of Education, Sinop University, Sinop, Turkey; b Department of Physics, Ondokuz Mayıs University, TR-55139 Samsun, Turkey; c Chemistry Programme, Denizli Higher Vocational School, Pamukkale University, TR-20159 Denizli, Turkey

## Abstract

In the mol­ecule of the title compound, C_14_H_12_FNO_2_, the aromatic rings are oriented at a dihedral angle of 48.17 (1)°. An intra­molecular O—H⋯N hydrogen bond results in the formation of a six-membered ring. The title mol­ecule is a phenol–imine tautomer, as evidenced by the C—O [1.351 (3) Å], C—N [1.282 (3) Å], and C—C [1.416 (3)–1.445 (3) Å] bond lengths. In the crystal, mol­ecules are linked by inter­molecular C—H⋯π inter­actions.

## Related literature

The present work is part of a structural study of Schiff bases, see: Özek *et al.* (2007 ▶); Odabaşoğlu *et al.* (2007 ▶); Albayrak *et al.* (2005 ▶). For related structures, see: Özek *et al.* (2007 ▶, 2009 ▶).
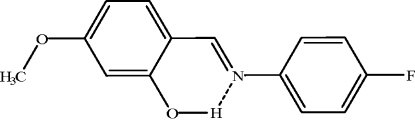


## Experimental

### 

#### Crystal data

C_14_H_12_FNO_2_
*M* *_r_* = 245.25
Monoclinic, 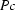

*a* = 13.1806 (7) Å
*b* = 7.1785 (5) Å
*c* = 6.4297 (3) Å
β = 97.967 (4)°
*V* = 602.49 (6) Å^3^
*Z* = 2
Mo *K*α radiation
μ = 0.10 mm^−1^
*T* = 296 K
0.68 × 0.48 × 0.17 mm

#### Data collection

Stoe IPDS II diffractometer
Absorption correction: integration (*X-RED32*; Stoe & Cie, 2002 ▶) *T*_min_ = 0.932, *T*_max_ = 0.985
6287 measured reflections
1399 independent reflections
1273 reflections with *I* > 2σ(*I*)
*R*_int_ = 0.037

#### Refinement

*R*[*F* ^2^ > 2σ(*F* ^2^)] = 0.035
*wR*(*F* ^2^) = 0.104
*S* = 1.09
1399 reflections
168 parameters
3 restraints
H atoms treated by a mixture of independent and constrained refinement
Δρ_max_ = 0.20 e Å^−3^
Δρ_min_ = −0.11 e Å^−3^

### 

Data collection: *X-AREA* (Stoe & Cie, 2002 ▶); cell refinement: *X-AREA*; data reduction: *X-RED32* (Stoe & Cie, 2002 ▶); program(s) used to solve structure: *SHELXS97* (Sheldrick, 2008 ▶); program(s) used to refine structure: *SHELXL97* (Sheldrick, 2008 ▶); molecular graphics: *ORTEP-3 for Windows* (Farrugia, 1997 ▶); software used to prepare material for publication: *WinGX* (Farrugia, 1999 ▶).

## Supplementary Material

Crystal structure: contains datablocks I, global. DOI: 10.1107/S1600536810000474/bt5163sup1.cif

Structure factors: contains datablocks I. DOI: 10.1107/S1600536810000474/bt5163Isup2.hkl

Additional supplementary materials:  crystallographic information; 3D view; checkCIF report

## Figures and Tables

**Table 1:** Hydrogen-bond geometry (Å, °) *Cg*1 and *Cg*2 are the centroids of C1—C6 and C9—C14 rings, respectively.

*D*—H⋯*A*	*D*—H	H⋯*A*	*D*⋯*A*	*D*—H⋯*A*
O1—H1⋯N1	0.82 (2)	1.87 (2)	2.615 (3)	150 (3)
C6—H6⋯*Cg*1^i^	0.93	2.73	3.4363	133
C11—H11⋯*Cg*2^ii^	0.93	2.93	3.6414	134
C14—H14⋯*Cg*2^iii^	0.93	2.91	3.6076	133

Symmetry codes: (i) 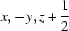; (ii) 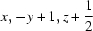; (iii) 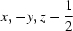.

**Fig. 1.**
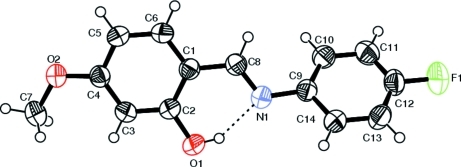
A view of (I), with the atom-numbering scheme. Dashed line indicates intramolecular hydrogen bond.

**Fig. 2.**
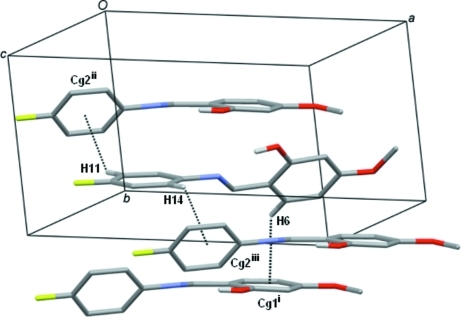
A partial packing diagram for (I), with C—H···Cg bonds shown as dashed lines. Cg1 and Cg2 are the centroids of C1—C6 and C9—C14 rings, respectively. Symmetry codes: (i) x, -y, z + 1/2; (ii) x, -y + 1, z + 1/2; (iii) x, -y, z - 1/2.

## supplementary crystallographic information

### Comment

The present work is part of a structural study of Schiff bases (Özek *et al.*, 2009; Özek *et al.*, 2007) and we report here the structure of (*E*)-2-(4-Fluorophenylimino)methyl-5-methoxyphenol, (I).

The *ortho*-hydroxy Schiff Bases that show tautomerism by the intramolecular proton transfer from an oxygen atom to the neighboring nitrogen atom are important compounds. These compounds can exist in three different structures as enol, keto or zwitterionic forms in the solid state. The title compound (I) consists of two aromatic rings (C1 to C6 and C9 to C14), and an imino frame (C9—N1—C8—C1). In (*E*)-2-(4-Fluorophenylimino)methyl- 5-methoxyphenol which adopts an E configuration about the C=N double bond, dihedral angle between the aromatic rings is 48.17 (1) °. The H atom in title compound (I) is located on atom O1, thus the phenol-imine tautomer is favored over the keto-amine form, as indicated by the C2—O1, C8—N1, C1—C8 and C1—C2 bond lengths (Fig. 1 and Table 2). The O1—C2 bond length of 1.351 (2) Å indicates single-bond character, whereas the N1—C8 bond length of 1.283 (2) Å indicates double-bond character. A similar work was observed for X-ray crystal and computational structural study of (*E*)-2-[(4-bromophenyl)iminomethyl] -4-methoxyphenol [C—O=1.358 (4) Å, C—N= 1.287 (4) Å, Özek *et al.*, 2007].

It is known that Schiff bases may exhibit thermochromism or photochromism, depending on the planarity or non-planarity of the molecule, respectively. Therefore, one can expect photochromic properties in (I) caused by non-planarity of the molecules; the dihedral angle between rings A(C1—C6) and B ring (C9—C14) is 48.17 (1) °. The intramolecular O—H···N hydrogen bond (Table 1) results in the formation of six-membered ring and it generates an S(6) ring motif. The O1···N1 distance of 2.614 (2) Å is comparable to those observed for analogous hydrogen bonds in "Three (*E*)-2-[(bromophenyl)iminomethyl]-4-methoxyphenols" [2.603 (2) Å, 2.638 (7) Å, 2.577 (4) Å; Özek *et al.*, 2007]. In the crystal structure, C—H···π interactions exist (Table 1) (Fig. 2).

### Experimental

The compound (*E*)-2-(4-Fluorophenylimino)methyl-5-methoxyphenol was prepared by reflux a mixture of a solution containing 4-methoxysalicylaldehyde (0.5 g 3.3 mmol) in 20 ml e thanol and a solution containing 4-fluoroaniline (0.37 g 3.3 mmol) in 20 ml e thanol. The reaction mixture was stirred for 1 h under reflux. The crystals of (*E*)-2-(4-Fluorophenylimino)methyl-5-methoxyphenol suitable for X-ray analysis were obtained from ethanol by slow evaporation (yield % 82; m.p. 368–369 K).

### Refinement

All H atoms except the hydroxyl H atom (which was freely refined) were refined using riding model with C—H distances of 0.96 Å for the methyl group and 0.93 Å for other H atoms. The displacement parameters of these H atoms were fixed at 1.2 *U*_eq_ of their parent carbon atom or 1.5 *U*_eq_ for the methyl group. The absolute structure could not be determined, and 1150 Friedel pairs were averaged before the last refinement.

### Crystal data

**Table:**

C_14_H_12_FNO_2_	*F*(000) = 256
*M**_r_* = 245.25	*D*_x_ = 1.352 Mg m^−^^3^
Monoclinic, *P**c*	Mo *K*α radiation, λ = 0.71073 Å
Hall symbol: P -2yc	Cell parameters from 11108 reflections
*a* = 13.1806 (7) Å	θ = 1.6–28.0°
*b* = 7.1785 (5) Å	µ = 0.10 mm^−^^1^
*c* = 6.4297 (3) Å	*T* = 296 K
β = 97.967 (4)°	Plate, yellow
*V* = 602.49 (6) Å^3^	0.68 × 0.48 × 0.17 mm
*Z* = 2	

### Data collection

**Table:**

Stoe IPDS II diffractometer	1399 independent reflections
Radiation source: fine-focus sealed tube	1273 reflections with *I* > 2σ(*I*)
plane graphite	*R*_int_ = 0.037
Detector resolution: 6.67 pixels mm^-1^	θ_max_ = 27.6°, θ_min_ = 2.8°
ω–scan rotation method	*h* = −17→17
Absorption correction: integration (*X-RED32*; Stoe & Cie, 2002)	*k* = −9→9
*T*_min_ = 0.932, *T*_max_ = 0.985	*l* = −8→8
6287 measured reflections	

### Refinement

**Table:**

Refinement on *F*^2^	Secondary atom site location: difference Fourier map
Least-squares matrix: full	Hydrogen site location: inferred from neighbouring sites
*R*[*F*^2^ > 2σ(*F*^2^)] = 0.035	H atoms treated by a mixture of independent and constrained refinement
*wR*(*F*^2^) = 0.104	*w* = 1/[σ^2^(*F*_o_^2^) + (0.0672*P*)^2^ + 0.0142*P*] where *P* = (*F*_o_^2^ + 2*F*_c_^2^)/3
*S* = 1.09	(Δ/σ)_max_ < 0.001
1399 reflections	Δρ_max_ = 0.20 e Å^−^^3^
168 parameters	Δρ_min_ = −0.11 e Å^−^^3^
3 restraints	Extinction correction: *SHELXL97* (Sheldrick, 2008), Fc^*^=kFc[1+0.001xFc^2^λ^3^/sin(2θ)]^-1/4^
Primary atom site location: structure-invariant direct methods	Extinction coefficient: 0.022 (7)

### Special details

**Table:**

Experimental. 237 frames, detector distance = 100 mm
Geometry. All e.s.d.'s (except the e.s.d. in the dihedral angle between two l.s. planes) are estimated using the full covariance matrix. The cell e.s.d.'s are taken into account individually in the estimation of e.s.d.'s in distances, angles and torsion angles; correlations between e.s.d.'s in cell parameters are only used when they are defined by crystal symmetry. An approximate (isotropic) treatment of cell e.s.d.'s is used for estimating e.s.d.'s involving l.s. planes.
Refinement. Refinement of *F*^2^ against ALL reflections. The weighted *R*-factor *wR* and goodness of fit *S* are based on *F*^2^, conventional *R*-factors *R* are based on *F*, with *F* set to zero for negative *F*^2^. The threshold expression of *F*^2^ > σ(*F*^2^) is used only for calculating *R*-factors(gt) *etc*. and is not relevant to the choice of reflections for refinement. *R*-factors based on *F*^2^ are statistically about twice as large as those based on *F*, and *R*- factors based on ALL data will be even larger.

### Fractional atomic coordinates and isotropic or equivalent isotropic displacement parameters (Å^2^)

**Table:**

	*x*	*y*	*z*	*U*_iso_*/*U*_eq_	
C1	0.67815 (17)	0.7844 (3)	0.5679 (3)	0.0430 (5)	
C2	0.68722 (16)	0.7118 (3)	0.3666 (3)	0.0452 (5)	
C3	0.78232 (18)	0.6912 (3)	0.3009 (4)	0.0473 (5)	
H3	0.7878	0.6379	0.1711	0.057*	
C4	0.86907 (16)	0.7504 (3)	0.4299 (3)	0.0448 (5)	
C5	0.86142 (16)	0.8247 (3)	0.6292 (3)	0.0482 (5)	
H5	0.9199	0.8648	0.7153	0.058*	
C6	0.76785 (16)	0.8378 (3)	0.6958 (3)	0.0472 (5)	
H6	0.7637	0.8834	0.8297	0.057*	
C7	0.9809 (2)	0.6644 (5)	0.1854 (5)	0.0735 (7)	
H7A	0.9579	0.5374	0.1792	0.088*	
H7B	1.0525	0.6683	0.1720	0.088*	
H7C	0.9429	0.7339	0.0730	0.088*	
C8	0.58033 (17)	0.7980 (3)	0.6444 (3)	0.0465 (5)	
H8	0.5785	0.8356	0.7822	0.056*	
C9	0.40316 (17)	0.7582 (3)	0.6157 (4)	0.0456 (5)	
C10	0.39603 (19)	0.6839 (3)	0.8123 (4)	0.0539 (5)	
H10	0.4544	0.6382	0.8942	0.065*	
C11	0.3028 (2)	0.6775 (4)	0.8871 (4)	0.0606 (6)	
H11	0.2976	0.6270	1.0184	0.073*	
C12	0.21810 (19)	0.7470 (4)	0.7637 (5)	0.0598 (6)	
C13	0.22135 (19)	0.8207 (4)	0.5678 (4)	0.0607 (6)	
H13	0.1625	0.8667	0.4877	0.073*	
C14	0.31454 (17)	0.8245 (4)	0.4932 (4)	0.0522 (5)	
H14	0.3185	0.8717	0.3599	0.063*	
N1	0.49593 (14)	0.7598 (2)	0.5277 (3)	0.0485 (5)	
O1	0.60343 (14)	0.6613 (3)	0.2332 (3)	0.0631 (5)	
O2	0.96531 (13)	0.7433 (3)	0.3800 (3)	0.0561 (4)	
F1	0.12629 (15)	0.7419 (3)	0.8381 (4)	0.0922 (6)	
H1	0.553 (2)	0.676 (4)	0.294 (5)	0.079 (10)*	

### Atomic displacement parameters (Å^2^)

**Table:**

	*U*^11^	*U*^22^	*U*^33^	*U*^12^	*U*^13^	*U*^23^
C1	0.0418 (10)	0.0416 (11)	0.0453 (12)	−0.0015 (8)	0.0053 (8)	−0.0006 (8)
C2	0.0420 (11)	0.0492 (11)	0.0433 (12)	−0.0009 (8)	0.0020 (9)	−0.0016 (8)
C3	0.0479 (11)	0.0539 (12)	0.0406 (10)	−0.0011 (9)	0.0075 (8)	−0.0044 (9)
C4	0.0433 (11)	0.0459 (10)	0.0459 (12)	0.0014 (8)	0.0083 (9)	0.0045 (9)
C5	0.0439 (11)	0.0547 (11)	0.0443 (11)	−0.0041 (8)	−0.0003 (9)	−0.0031 (9)
C6	0.0493 (12)	0.0510 (11)	0.0403 (11)	−0.0007 (8)	0.0034 (9)	−0.0047 (8)
C7	0.0540 (14)	0.109 (2)	0.0612 (15)	−0.0001 (14)	0.0216 (11)	−0.0148 (14)
C8	0.0456 (11)	0.0472 (11)	0.0467 (11)	0.0012 (8)	0.0068 (9)	−0.0022 (8)
C9	0.0427 (11)	0.0453 (11)	0.0492 (12)	−0.0012 (8)	0.0073 (9)	−0.0028 (8)
C10	0.0521 (12)	0.0561 (12)	0.0527 (13)	0.0052 (10)	0.0051 (10)	0.0028 (10)
C11	0.0668 (16)	0.0630 (13)	0.0541 (13)	−0.0032 (12)	0.0158 (12)	0.0027 (11)
C12	0.0453 (13)	0.0678 (14)	0.0692 (17)	−0.0077 (10)	0.0184 (12)	−0.0090 (12)
C13	0.0435 (12)	0.0705 (15)	0.0663 (17)	−0.0007 (11)	0.0008 (11)	−0.0035 (12)
C14	0.0461 (12)	0.0593 (12)	0.0503 (13)	−0.0003 (9)	0.0041 (9)	0.0013 (10)
N1	0.0415 (10)	0.0532 (10)	0.0509 (11)	0.0016 (8)	0.0063 (8)	−0.0004 (8)
O1	0.0438 (8)	0.0922 (12)	0.0515 (9)	−0.0082 (8)	0.0007 (7)	−0.0194 (9)
O2	0.0420 (8)	0.0746 (12)	0.0529 (9)	−0.0021 (7)	0.0108 (7)	−0.0033 (8)
F1	0.0568 (10)	0.1257 (16)	0.1005 (15)	−0.0092 (10)	0.0334 (9)	−0.0025 (11)

### Geometric parameters (Å, °)

**Table:**

C1—C6	1.397 (3)	C8—N1	1.282 (3)
C1—C2	1.416 (3)	C8—H8	0.9300
C1—C8	1.445 (3)	C9—C10	1.387 (3)
C2—O1	1.351 (3)	C9—C14	1.399 (3)
C2—C3	1.385 (3)	C9—N1	1.417 (3)
C3—C4	1.383 (3)	C10—C11	1.381 (3)
C3—H3	0.9300	C10—H10	0.9300
C4—O2	1.352 (3)	C11—C12	1.371 (4)
C4—C5	1.404 (3)	C11—H11	0.9300
C5—C6	1.364 (3)	C12—F1	1.362 (3)
C5—H5	0.9300	C12—C13	1.372 (4)
C6—H6	0.9300	C13—C14	1.379 (3)
C7—O2	1.414 (3)	C13—H13	0.9300
C7—H7A	0.9600	C14—H14	0.9300
C7—H7B	0.9600	O1—H1	0.824 (19)
C7—H7C	0.9600		
			
C6—C1—C2	117.86 (19)	N1—C8—C1	121.93 (19)
C6—C1—C8	120.22 (19)	N1—C8—H8	119.0
C2—C1—C8	121.89 (18)	C1—C8—H8	119.0
O1—C2—C3	118.2 (2)	C10—C9—C14	119.1 (2)
O1—C2—C1	120.96 (19)	C10—C9—N1	122.6 (2)
C3—C2—C1	120.86 (18)	C14—C9—N1	118.2 (2)
C4—C3—C2	119.5 (2)	C11—C10—C9	120.4 (2)
C4—C3—H3	120.3	C11—C10—H10	119.8
C2—C3—H3	120.3	C9—C10—H10	119.8
O2—C4—C3	124.8 (2)	C12—C11—C10	118.6 (3)
O2—C4—C5	114.78 (19)	C12—C11—H11	120.7
C3—C4—C5	120.4 (2)	C10—C11—H11	120.7
C6—C5—C4	119.69 (19)	F1—C12—C11	118.6 (3)
C6—C5—H5	120.2	F1—C12—C13	118.4 (3)
C4—C5—H5	120.2	C11—C12—C13	123.0 (2)
C5—C6—C1	121.6 (2)	C12—C13—C14	118.0 (2)
C5—C6—H6	119.2	C12—C13—H13	121.0
C1—C6—H6	119.2	C14—C13—H13	121.0
O2—C7—H7A	109.5	C13—C14—C9	120.8 (2)
O2—C7—H7B	109.5	C13—C14—H14	119.6
H7A—C7—H7B	109.5	C9—C14—H14	119.6
O2—C7—H7C	109.5	C8—N1—C9	119.66 (17)
H7A—C7—H7C	109.5	C2—O1—H1	108 (3)
H7B—C7—H7C	109.5	C4—O2—C7	118.77 (19)
			
C6—C1—C2—O1	−178.6 (2)	C14—C9—C10—C11	−0.7 (3)
C8—C1—C2—O1	3.5 (3)	N1—C9—C10—C11	−176.9 (2)
C6—C1—C2—C3	1.3 (3)	C9—C10—C11—C12	−0.4 (4)
C8—C1—C2—C3	−176.6 (2)	C10—C11—C12—F1	−179.5 (2)
O1—C2—C3—C4	176.8 (2)	C10—C11—C12—C13	0.9 (4)
C1—C2—C3—C4	−3.1 (3)	F1—C12—C13—C14	−179.7 (2)
C2—C3—C4—O2	−177.68 (19)	C11—C12—C13—C14	−0.1 (4)
C2—C3—C4—C5	2.3 (3)	C12—C13—C14—C9	−1.1 (4)
O2—C4—C5—C6	−179.79 (19)	C10—C9—C14—C13	1.5 (3)
C3—C4—C5—C6	0.2 (3)	N1—C9—C14—C13	177.9 (2)
C4—C5—C6—C1	−2.1 (3)	C1—C8—N1—C9	174.01 (17)
C2—C1—C6—C5	1.3 (3)	C10—C9—N1—C8	−40.8 (3)
C8—C1—C6—C5	179.26 (19)	C14—C9—N1—C8	143.0 (2)
C6—C1—C8—N1	176.2 (2)	C3—C4—O2—C7	−2.2 (3)
C2—C1—C8—N1	−5.9 (3)	C5—C4—O2—C7	177.8 (2)

### Hydrogen-bond geometry (Å, °)

**Table:**

*Cg*1 and *Cg*2 are the centroids of C1—C6 and C9—C14 rings, respectively.

**Table:**

*D*—H···*A*	*D*—H	H···*A*	*D*···*A*	*D*—H···*A*
O1—H1···N1	0.82 (2)	1.87 (2)	2.615 (3)	150 (3)
C6—H6···Cg1^i^	0.93	2.73	3.4363	133
C11—H11···Cg2^ii^	0.93	2.93	3.6414	134
C14—H14···Cg2^iii^	0.93	2.91	3.6076	133

Symmetry codes: (i) *x*, −*y*, *z*+1/2; (ii) *x*, −*y*+1, *z*+1/2; (iii) *x*, −*y*, *z*−1/2.
